# Telemedicine and Alzheimer’s Disease From Studio-Based Videoconferencing to Mobile Handheld Cell Phones

**DOI:** 10.4137/jcnsd.s2296

**Published:** 2009-03-30

**Authors:** PK (Poh-Kooi) Loh

**Affiliations:** Department of Geriatric Medicine, Royal Perth Hospital, Wellington Street, Perth, Western Australia 6000.

**Keywords:** Alzheimer’s, telemedicine, information and communication technology

## Abstract

The use of Telemedicine in the assessment of cognitive impairment and Alzheimer’s Disease is evolving with advances in Information and Communication Technology. This article outlines the course of evolution in Western Australia, a large state with a sparse population outside of the capital city. The starting point of the evolution, video-conferencing in Telehealth studios, is reviewed as well as the next stage, use of desktop technology, which enables the physician to consult from the office or clinic. A pilot study currently being undertaken to assess the validity of the latest stage in evolution of Telehealth—the use of handheld mobile cell phone video calling that allows the physician and patient to interact at locations convenient to both parties. The pitfalls and implications of the use of this stage, should it prove to be a valid approach, are discussed.

## Introduction

Advances in Information and Communication Technology (ICT) have permitted the evolution of Telemedicine in the assessment of cognitive impairment and Alzheimer’s Disease (AD). This article describes the course of this evolution in Western Australia (WA). Telemedicine involves the transmission of images, voice and data between two or more sites using telecommunications and ICT to provide health services such as clinical advice, consultation, education and training.^1^ Within this article the term Telemedicine is specifically used when Telehealth is utilised for clinically-oriented medical applications rather than other types of health services such as patient support services and medical administration. Telemedicine has obvious application in WA, a large state of 2.16 million inhabitants in which non-city dwellers, of which there are at least five hundred thousand, are sparsely populated.^2^ Given the anticipated increase in the number of people living with AD and the associated cost to the community, the capability to assess, manage and follow-up as well as study individuals with cognitive impairment in rural WA is crucial. However, this capability is limited by reduced access to medical specialists and other health professionals. Telemedicine offers a means of overcoming this limitation by providing services from a distance. As such it substantially decreases the burden on patients and their families who would otherwise have to make multiple trips over long distances to attend specialist appointments.

The diagnosis of AD, treatment and monitoring of therapy requires numerous visits to the specialist physician. Once diagnosed, there is specific pharmacological therapy available for AD in Australia, which includes Acetyl Cholinesterase Inhibitors (ACI) and Memantine a N-methyl-D-aspartate (NMDA) receptor antagonist.^3^ The use of ACI and Memantine are restricted by specialist clinician assessment. In Australia pharmaceuticals are available on receipt of a nominal patient copayment from the national formulary of medicines the Prescribing Benefit Schedule (PBS). The PBS restrictions for AD involve the provision of an authority prescription on the Prescribing Benefit Schedule. Subsidised prescriptions can only be provided if patients’ have mild to moderate AD with a Standardised Mini Mental State (SMMSE) score of 10 to 24 for ACI and 10 to 14 on SMMSE for Memantine. Patients with SMMSE scores outside of the above range do not have access to government subsidised AD medications available on the PBS. These prescriptions need to be approved by a specialist practitioner such as a neurologist, psychiatrist or geriatrician who has made the diagnosis. The specialist physician reviews the patient after 3 months of therapy and there must be at least a two point increase on the SMMSE score to be able to access ongoing prescriptions of the treatment on subsidised PBS prescriptions. Due to the inequity in distribution of the physician workforce in WA, this specialist assessment is not readily available to patients in rural WA. Patients have to travel long distances or physicians have to travel to rural areas. Telemedicine provides a potential solution to this problem by allowing specialists based in capital cities to assess and diagnose patients in areas outside of metropolitan health institutions. The assessment of cognitive impairment, the diagnosis of AD, followed by potential for management of AD at a distance using Telemedicine is reported here.

## Background

The use of Telemedicine for assessing cognitive impairment in WA began with studio-based videoconferencing. The physician and patient had to travel to their respective studios where selected validated psychological assessment instruments were used for assessments. A pilot study demonstrated that the use of cognitive assessment tools such as the SMMSE and Geriatric Depression Scale (GDS) were valid and reliable via Telemedicine.^4^ This was followed by the development of a protocol to assess AD via studio based Telemedicine videoconferencing. This protocol demonstrated that there was high concordance (kappa 0.8) between the diagnosis of AD performed face-to-face and via Telemedicine.^5^ The studio-based technology was bought-off-the shelf and consisted of a VCON cruiser, version 4.0, videoconferencing unit with Sony D31 PTZ camera at both the receiving and transmitting studios (Vcon, Polycom, California, U.S.A; Camera from Sony Corporation, Tokyo, Japan). A range of transmission bandwidths (128 kilobits per second (kbps), 256 kbps and 384 kbps) were used. This was followed by videoconferencing utilising Internet broadband with an internet video camera and a desktop personal computer. The use of Telemedicine utilising desktop computer based videoconferencing equipment allowed the physician to conduct assessments from the office but the patient still had to travel to the studio. The desktop computer peripherals for videoconferencing such as the web camera and audio equipment were from Logitech (Apples, Switzerland) also bought off-the-shelf.

Other studies also indicate that Telemedicine is a suitable modality for distance assessment of cognitive impairment and AD.^6^–^10^ These studies found that there was no significant difference between Telemhealth and face-to-face assessments. Telemedicine was also suitable for cognitive intervention in AD.^11^ ICT and Telemedicine are already in use for managing heart failure and diabetes for older people.^12^ With the evolving technology, the next step in the development of Telemedicine AD assessment protocols is the use of handheld mobile cell phones (HMC) video calling for assessment, followed by its use in management and follow-up of patients with cognitive impairment if the HMC medium of assessment is appropriate.

**Figure 1:**
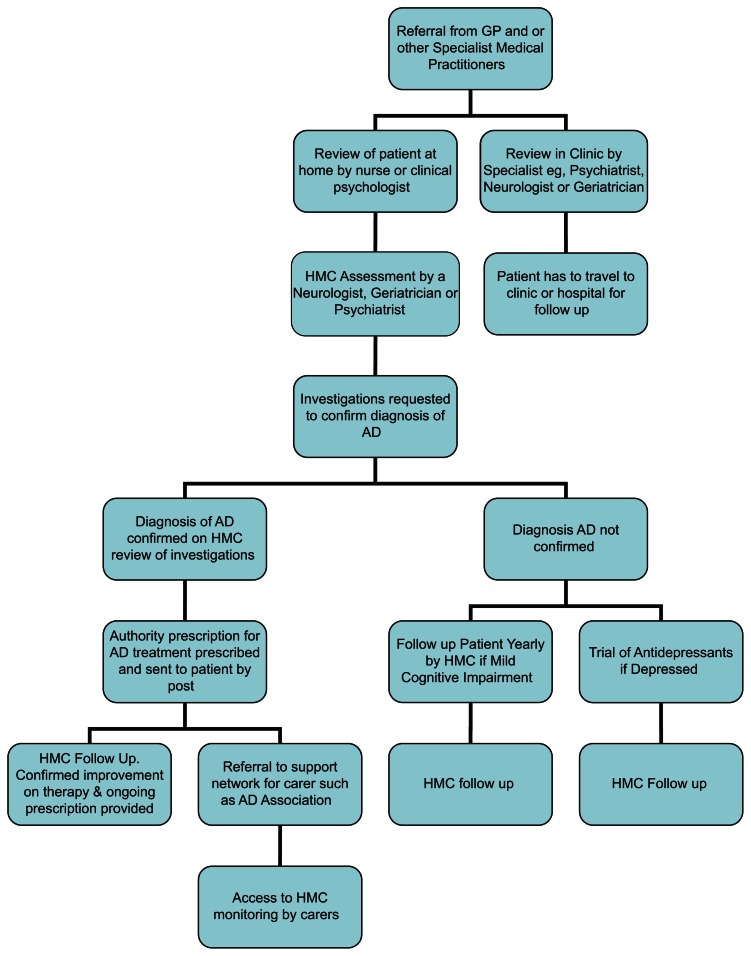
Handheld Mobile Cell phone (HMC) patient flow chart in Alzheimer’s Disease.

Figure 1 illustrates the possible use HMC in AD and may save the patients numerous trips to the clinic. This may alleviate the care giver burden on relatives and carers.

## Handheld Mobile Cell Phones (HMC) Pitfalls

The use of HMC is currently a novel approach of providing assessment and treatment of cognitive impairment to individuals in rural WA. Conventional Telemedicine studios located in health facilities throughout country WA are not required and assessments can be performed in the home of the patient. The use of HMC in rural areas is limited by the distance the wireless networks can carry the video calls. The use of HMC in AD is not new but was mainly used for monitoring patients. There are services in North America that allow relatives of patients with AD to monitor the patients with their HMC using video telephone calls.^13^ Other uses of HMC include giving patients with AD cell phones that contain global positioning satellite (GPS) devices that allow easy location of the patient and allows the patient to call for help if lost.^14^ However, there is scant literature on the use of HMC as a tool for assessing AD by a clinician.

In WA, researchers are currently trying to develop a pilot protocol for HMC assessment of AD. The development of the protocol itself has been a challenge because of the large variety of devices that are available on the market. A selection of HMCs are being evaluated for assessment of AD using off-the-shelf units that are purchased from retail outlets. Some devices require a financial account with a cell phone carrier similar to AT&T (Dallas, Texas, U.S.A.) or Verizon Wireless (Basking Ridge, New Jersey, U.S.A.) in North America with the Australian cell phone carriers being: Telstra, Optus, Vodaphone, and Virgin Mobile to name a few. Some of the devices being tested do not require an account with a carrier and provide cell phone services on a pay as you go basis. Pay as you go HMC maybe an alternative for those who are not able to sustain a financial agreement with the cell phone carrier. The HMC networks are not consistent and calls can suddenly stop as the device drops out of the network and the call terminates. Furthermore, within some hospitals, clinics or health facilities there are areas where transmission by HMC is not possible because of disruption to sensitive medical devices or special shielding in the building or blind spots in the network. The HMC have a variety of cameras, screen sizes and audio quality with the suitability being determined by trial and error by researchers.

To try and pick the best network and HMC, the research team has commenced testing the devices and 15 patients have been assessed with a variety of cognitive assessment instruments and HMC. The trial principles for the assessment and management of cognitive impairment performed via studio-based videoconferencing and HMC video phone calls are similar. All patients referred for assessment of cognitive function and treatment of AD are randomized to standardized dementia assessment via one of the two media for assessment. Patients have a face-to-face assessment for AD followed by HMC video call assessment or vice versa. The initial medium of assessment is randomized in order to minimize possible bias from period and order effects. Assessors are blinded to results from assessments by different media and other investigators. The subjects are patients in aged care facilities, hospital wards or clinics where there are subjects prepared to be assessed by Telemedicine videoconferencing. Patients in clinics mainly live in the community in supported facilities with long term care or they maybe living independently with home supports. All subjects and their healthcare providers were provided written informed consent. Inclusion criteria are any patient or volunteer, above 50 years old without impaired hearing or visual acuity and prepared to accept informed consent. Exclusion criteria include any patients unable to understand spoken or written English sufficiently to complete AMTS or SMMSE in English. Also excluded from the pilot study are any patients with speech impediment or dysphasia or patients with SMMSE score less than 10.

From the test already conducted the actual cell phone transmission time utilising abbreviated cognitive assessment instruments last about 5 to 10 minutes. There have been no complaints about quality of the devices by the patients. Surprisingly all the patients were extremely enthusiastic about the use of the cell phones as a modality for AD. The results of the cognitive assessments are not publishable because different HMC are being used to determine a workable protocol for a pilot study that can then be published.

## Discussion

It is important to assess the use of HMC Telemedicine Video Calling for Cognitive Assessment in rural WA. If there are negative results there is evidence to reduce further input of resources into another medium of ICT in Telemedicine, which has minimal benefit to the community. If there are positive results it will provide evidence to support convenient services and health prevention strategies, which will benefit patients who live in rural and remote areas.

The use of HMC assessments would supplement the current services provided face-to-face and via Telemedicine studios. There are 75 out of a total of 104 studios in WA current in use^15^ for Telemedicine in WA. Health professionals and Aged Care Assesment Teams (ACAT) currently conducting cognitive assessments should have access to HMC to allow them to more conveniently assess their patients. ACATs are federally-funded community-based multidisciplinary teams that provide assessment and support services for older people with cognitive impairment in Australia.^16^ A formal costing of distributing HMC to ACAT, patients or their families for AD has not been performed to date.

Benefits to the individual in rural communities include more convenient access to specialist treatment. HMC also allow access to health improvement and support programs for dementia and AD related depression from the home via the video calling enabled cell phones. These strategies promote prevention of disability from cognitive impairment or related illnesses such as behavioural problems and depression with patients having to travel less. In comparison to studio-based Telemedicine greater number of patients will receive expert cognitive assessment because of the relative low cost of hand held units and availability of prepaid wireless cell phone services. The benefits to the health system include more equitable distribution of specialist medical manpower. Decreased time and resources spent on transportation may improve delivery of AD assessments and treatments to rural areas. These benefits may also apply to patients living in metropolitan suburban areas who have difficulty accessing specialist care due to the traffic and transportation difficulties.

The benefits to medical research include opportunities to offer clinical trial participation to remote sites via the HMC and increase in the number of patients enrolled in promising dementia management strategies. More so with the recent proposals to changes in the criteria for diagnosing AD where medical imaging is required.^17^ ICT and Telemedicine will allow rapid combination of Teleradiology and distance clinical assessment by the physician.
